# Crystal structure and Hirshfeld surface analysis of 3-[(*E*)-2-(2-bromo-4,5-di­meth­oxy­phen­yl)ethen­yl]-5,5-di­methyl­cyclo­hex-2-en-1-one

**DOI:** 10.1107/S2056989025006413

**Published:** 2025-07-23

**Authors:** Muruganandham Rajkumar, Uthirapathi Rajapandiyan, Haridoss Manikandan, Velusamy Rajathi, Sivashanmugam Selvanayagam

**Affiliations:** a Department of Chemistry Annamalai University, Annamalainagar Chidambaram 608 002 India; b PG & Research Department of Zoology, Government Arts College, C Mutlur, Chidambaram 608 102, India; c PG & Research Department of Physics, Government Arts College, Melur 625 106, India; Vienna University of Technology, Austria

**Keywords:** cyclo­hexene derivatives, inter­molecular hydrogen bonds, Hirshfeld surface analysis, crystal structure

## Abstract

In the title compound, C_18_H_21_BrO_3_, which represents an isophorone (3,5,5-trimethyl-2-cyclo­hexen-1-one) derivative, the cyclo­hexene ring adopts a twist-boat conformation. An intra­molecular C—H⋯Br hydrogen bond between a methine H atom and the Br substituent at the phenyl ring leads to the stabilization of the mol­ecular conformation. Inter­molecular C—H⋯O hydrogen bonds as well as π–π inter­actions are observed in the crystal. The inter­molecular inter­actions were qu­anti­fied and analysed using Hirshfeld surface analysis, revealing that H⋯H inter­actions contribute most (46.9%) to the crystal packing.

## Abstract 2

The crystal packing of the title compound, C_18_H_21_BrO_3_, is consolidated by C—H⋯O hydrogen bonds, which form inversion dimers with *R*_2_^2^(24) graph-set motifs.

## 1. Chemical context

Isophorone (3,5,5-trimethyl-2-cyclo­hexen-1-one) is a colourless to pale yellow cyclic *α*,*β*-unsaturated ketone, characterized by a distinctive peppermint-like odour (Kataoka *et al.*, 2007 ▸). The presence of a conjugated enone system makes isophorone an excellent synthon for carbon–carbon bond-forming reactions, thus establishing its role as a valuable inter­mediate in synthetic organic chemistry. In recent years, isophorone-derived compounds have garnered considerable attention due to their broad spectrum of biological activities, including anti­cancer (Logeshwari *et al.*, 2024 ▸), anti­microbial and anti­oxidant (Kozak *et al.*, 2019 ▸) effects. These pharmacological properties are primarily attributed to the introduction of styryl or aryl moieties through condensation with bioactive aldehydes, positioning isophorone as an important scaffold in drug discovery.
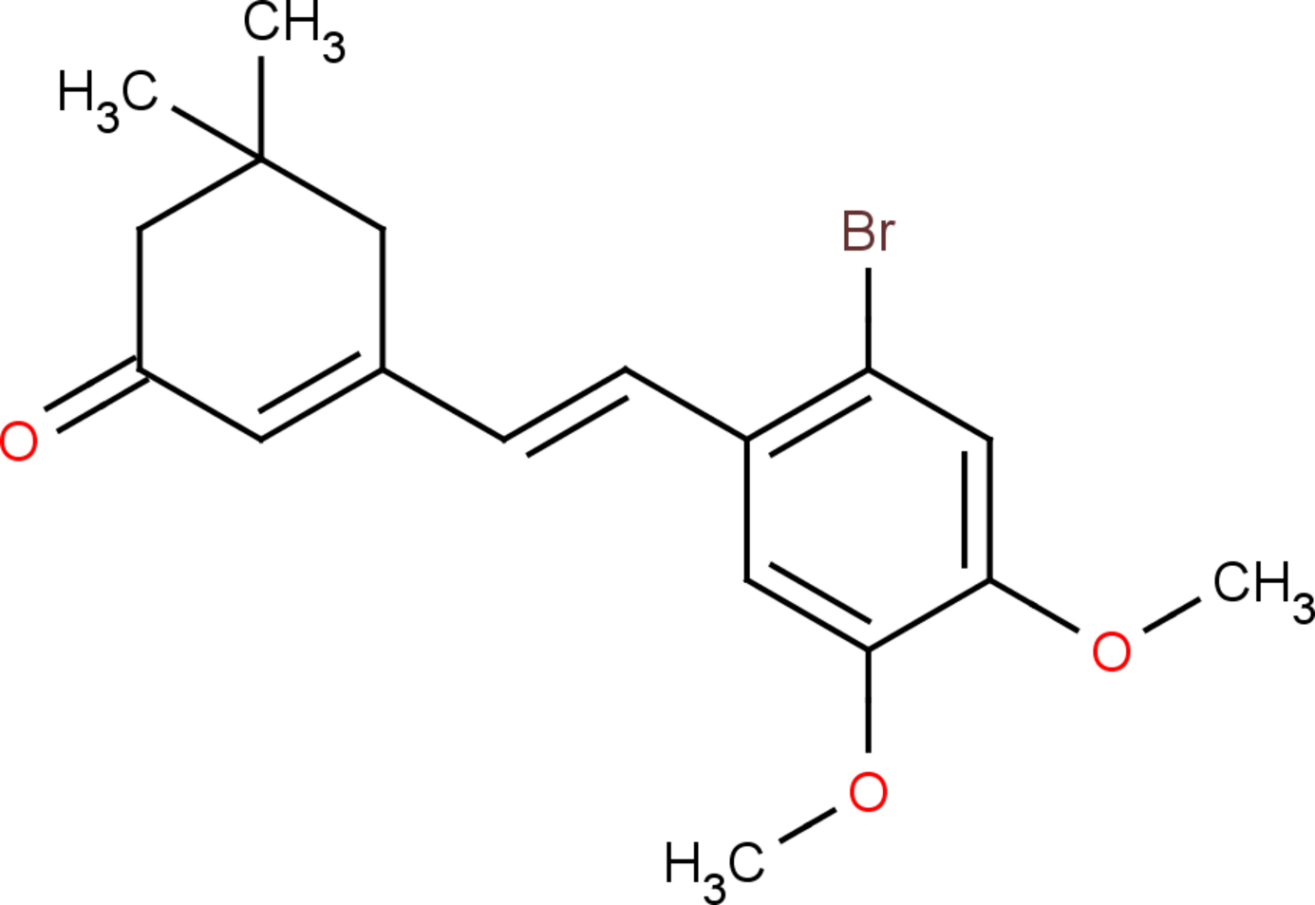


In the context given above, we synthesized an isophorone derivative and report here the mol­ecular and crystal structure, and Hirshfeld surface analysis of 3-[(*E*)-2-(2-bromo-4,5-di­meth­oxy­phen­yl)ethen­yl]-5,5-di­methyl­cyclo­hex-2-en-1-one, (I).

## 2. Structural commentary

**Figure 1:**
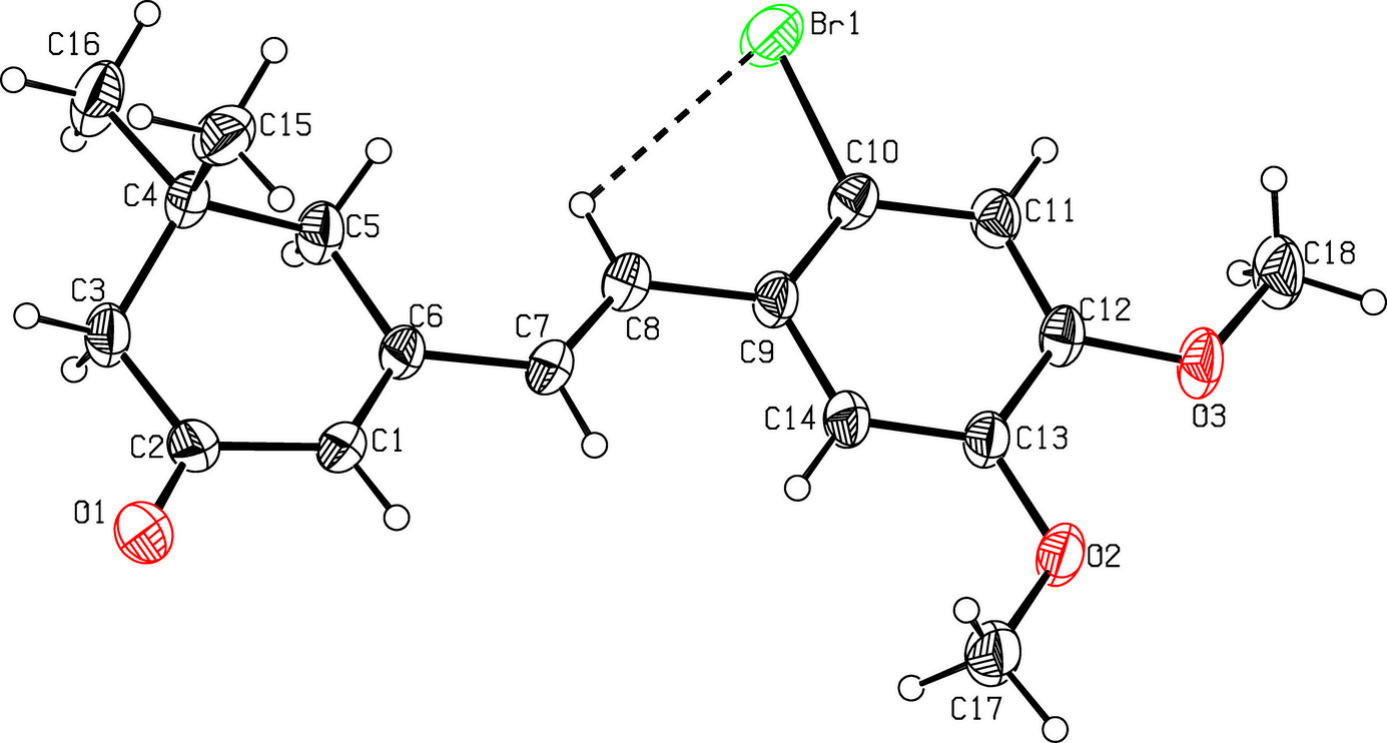
A view of the mol­ecular structure of compound (I), showing the atom labelling. Displacement ellipsoids are drawn at the 30% probability level. The intra­molecular hydrogen bond is shown as a dashed line.

The mol­ecular structure of (I) is displayed in Fig. 1 ▸. The O1—C2 [1.222 (3) Å], C1—C6 [1.342 (3) Å] and C7—C8 [1.322 (3) Å] bond lengths confirm the double-bond character. The reduction of the bond angle C10—C9—C14 [116.2 (2)°] is due to the short contact H7⋯H14 (2.05 Å). The cyclo­hexene ring adopts a twist-boat conformation with puckering parameters (Cremer & Pople, 1975 ▸) *q*_2_ = 0.374 (2) Å, *q*_3_ = −0.274 (2) Å, *Q*_T_ = 0.464 (2) Å and φ = 349.5 (4)°. Atom C4 deviates by −0.638 (2) Å from the least-squares plane through the remaining five atoms (C1–C3/C5/C6) of the ring. The mean plane calculation of the bromo dimethyl phenyl ring reveals that the methyl atoms C17 and C18 deviate by −0.051 (2) and 0.065 (2) Å, respectively, from the plane while the bromine atom deviates by 0.008 (1) Å. A weak intra­molecular contact (Table 1 ▸) between a methine H atom and the Br atom attached to the phenyl ring leads to the stabilization of the mol­ecular conformation. This C8—H8⋯Br1 inter­action forms an *S*(5) ring motif (Bernstein *et al.*, 1995 ▸), as shown in Fig. 1 ▸.

## 3. Supra­molecular features

**Figure 2:**
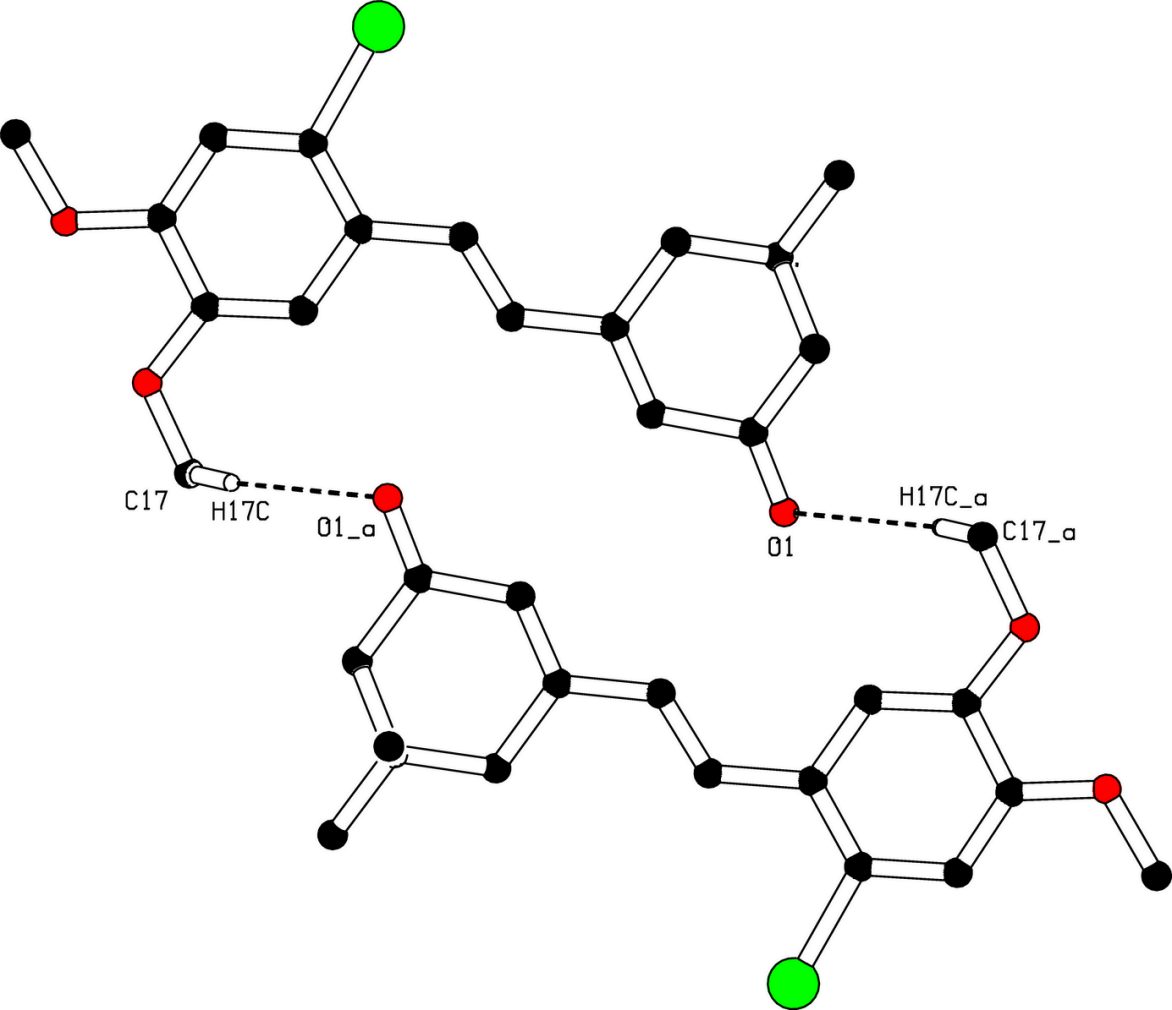
The formation of a centrosymmetric dimer in the crystal structure of (I) through C—H⋯O hydrogen bonds. [Symmetry code: (*a*) −*x* + 1, −*y* + 2, -*z.*]

**Figure 3:**
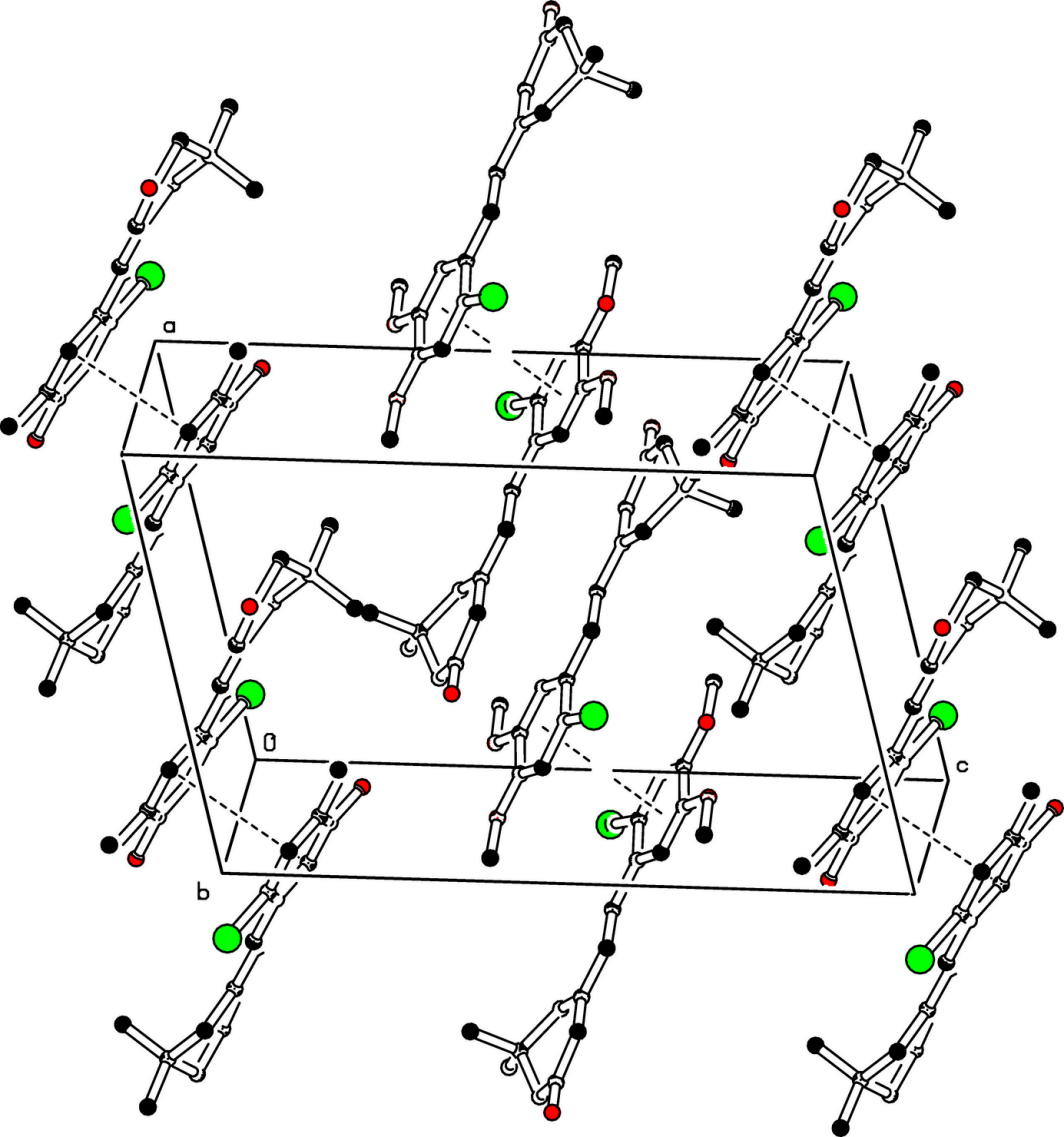
The crystal packing of (I) with π–π inter­molecular inter­actions shown as dashed lines. For clarity, H atoms have been omitted.

In the crystal, mol­ecules associate pairwise *via* C17—H17*C*⋯O1^i^ hydrogen bonds (Table 1 ▸) into inversion dimers with an 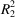
(24) graph-set motif (Etter *et al.*, 1990 ▸), as shown in Fig. 2 ▸. Moreover, π–π inter­actions are observed between the centroids of inversion-related benzene rings (C9–C14) with a centroid-to-centroid distance of 3.825 (1) Å and a slippage of 1.435 Å (Fig. 3 ▸).

## 4. Hirshfeld surface analysis

**Figure 4:**
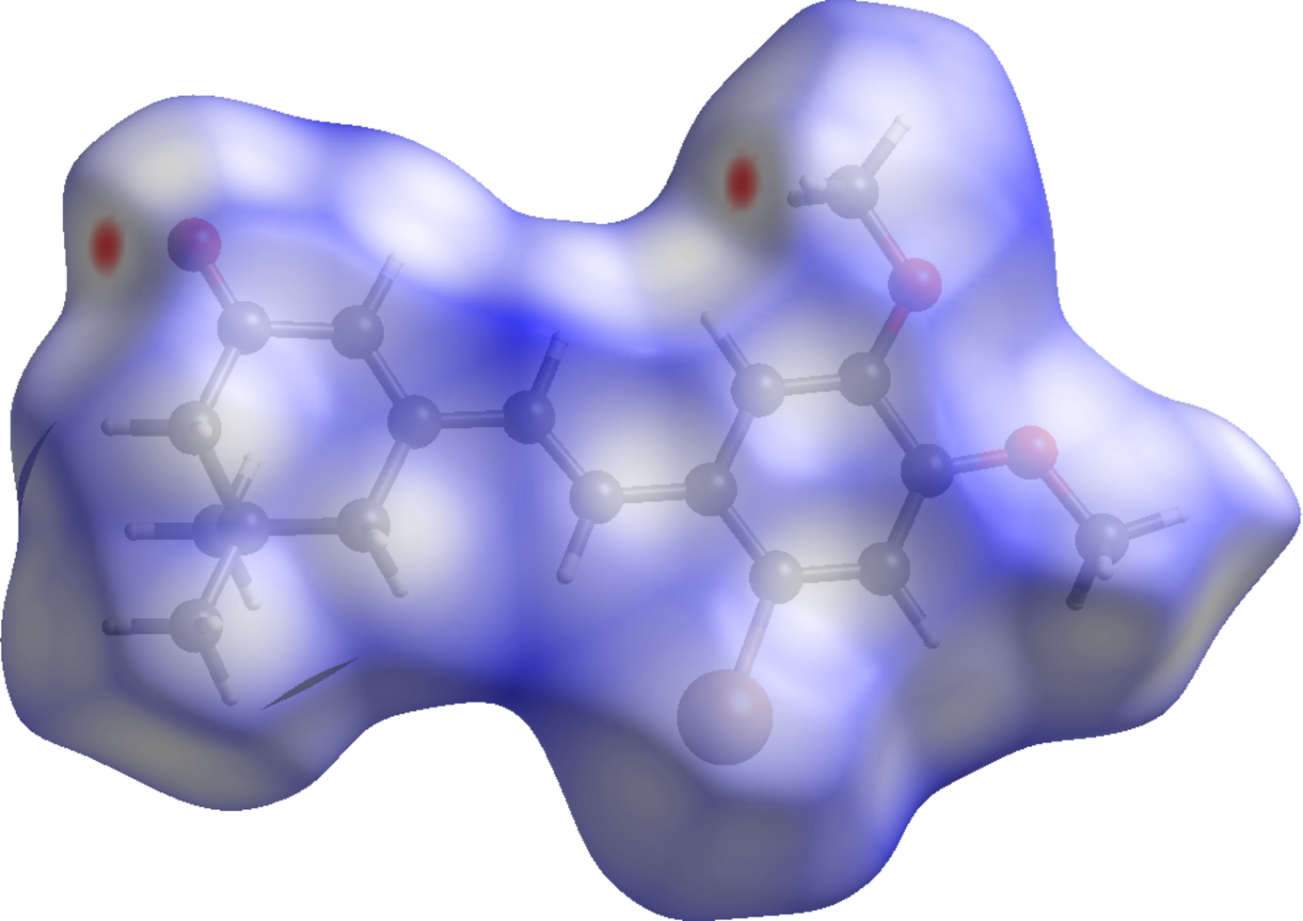
A view of the Hirshfeld surface mapped over *d*_norm_ for compound (I).

Inter­molecular inter­actions were qu­anti­fied by a Hirshfeld surface (HS) analysis (Spackman & Jayatilaka, 2009 ▸) using *CrystalExplorer* (Spackman *et al.*, 2021 ▸). The HS mapped over *d*_norm_ is illustrated in Fig. 4 ▸. where the deep-red spots at O1 and H17*C* represent distances shorter than van der Waals radii and are indicative of the inter­molecular C—H⋯O hydrogen bond discussed above.

**Figure 5:**
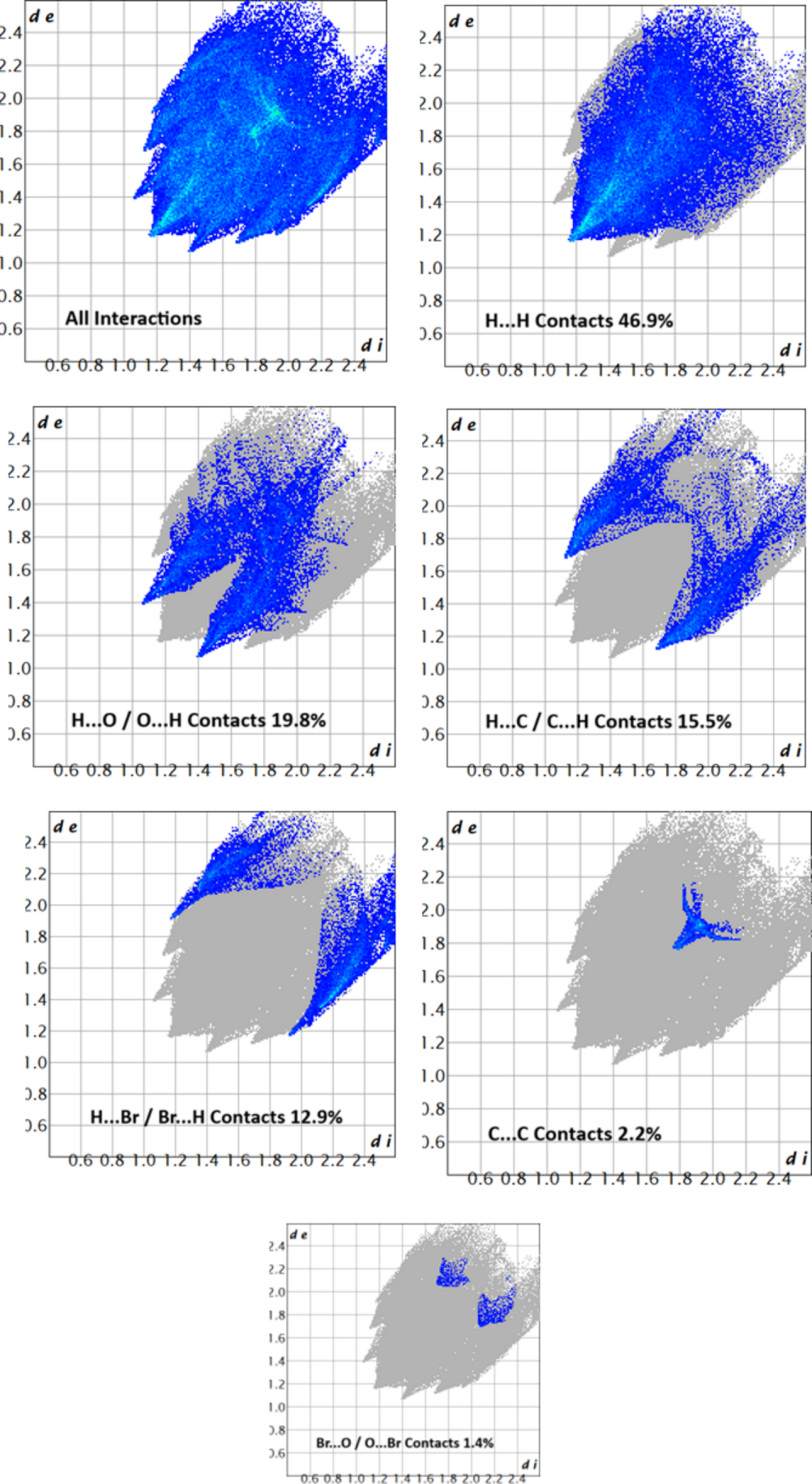
Two-dimensional fingerprint plots for compound (I), showing all inter­actions, and delineated into H⋯H, H⋯O/O⋯H, H⋯C/C⋯H, H⋯Br/Br⋯H, C⋯C and Br⋯O/O⋯Br inter­actions. The *d*_i_ and *d*_e_ values are the closest inter­nal and external distances (in Å) from given points on the Hirshfeld surface.

The associated two-dimensional fingerprint plots (McKinnon *et al.*, 2007 ▸) provide qu­anti­tative information about the non-covalent inter­actions in the crystal packing in terms of the percentage contribution of the inter­atomic contacts (Spackman & McKinnon, 2002 ▸). The overall two-dimensional fingerprint plot is shown in Fig. 5 ▸ (top left). The HS analysis reveals that H⋯H and H⋯O/O⋯H contacts are the main contributors to the crystal packing, followed by H⋯C/C⋯H, H⋯Br/Br⋯H, C⋯C and Br⋯O/O⋯Br contacts (Fig. 5 ▸).

## 5. Synthesis and crystallization

Compound (I) was synthesized by dissolving isophorone (1 mmol, 0.140 g) and 2-bromo-4,5-di­meth­oxy­benzaldehyde (1 mmol, 0.247 g) in absolute ethanol (15 ml) in a round-bottom flask of 50 ml and stirring. Following that, a 20%_wt_ sodium hydroxide solution (1 mmol, 0.04 g) was added dropwise under continuous stirring. The reaction mixture was then stirred at ambient temperature (298 K) for 6 h. The progress of the condensation reaction was monitored from time to time by thin-layer chromatography (TLC) on a hexa­ne–ethyl acetate (7:3) solvent system. After completion, the mixture was transferred to crushed ice, which caused the development of a yellow precipitate. The solid was filtered off under reduced pressure, washed with cold distilled water, and dried at room temperature. The crude product was recrystallized from ethanol to obtain crystals of (I).

## 6. Refinement

Crystal data, data collection and structure refinement details are summarized in Table 2 ▸. All H atoms were placed in idealized positions and allowed to ride on their parent atoms: C—H = 0.93–0.97 Å with *U*_iso_(H) = 1.5*U*_eq_(C) for methyl H atoms and *U*_iso_(H) = 1.2*U*_eq_(C) for other H atoms.

## Supplementary Material

Crystal structure: contains datablock(s) I, shelx. DOI: 10.1107/S2056989025006413/wm5763sup1.cif

Structure factors: contains datablock(s) I. DOI: 10.1107/S2056989025006413/wm5763Isup2.hkl

Supporting information file. DOI: 10.1107/S2056989025006413/wm5763Isup3.cml

CCDC reference: 2473698

Additional supporting information:  crystallographic information; 3D view; checkCIF report

## Figures and Tables

**Table 1:** Hydrogen-bond geometry (Å, °)

*D*—H⋯*A*	*D*—H	H⋯*A*	*D*⋯*A*	*D*—H⋯*A*
C8—H8⋯Br1	0.93	2.76	3.212 (2)	111
C17—H17*C*⋯O1^i^	0.96	2.58	3.495 (3)	159

Symmetry code: (i) 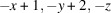.

**Table 2:** Experimental details

Crystal data	
Chemical formula	C_18_H_21_BrO_3_
*M*_r_	365.26
Crystal system, space group	Monoclinic, *P*2_1_/*c*
Temperature (K)	300
*a*, *b*, *c* (Å)	11.9084 (8), 8.1667 (5), 18.1737 (13)
β (°)	102.231 (2)
*V* (Å^3^)	1727.3 (2)
*Z*	4
Radiation type	Mo *K*α
μ (mm^−1^)	2.39
Crystal size (mm)	0.19 × 0.17 × 0.09

Data collection	
Diffractometer	Bruker APEXII CCD
Absorption correction	Multi-scan (*SADABS*; Krause *et al.*, 2015 ▸)
*T*_min_, *T*_max_	0.636, 0.746
No. of measured, independent and observed [*I* > 2σ(*I*)] reflections	33400, 4282, 2767
*R*_int_	0.045
(sin θ/λ)_max_ (Å^−1^)	0.667

Refinement	
*R*[*F*^2^ > 2σ(*F*^2^)], *wR*(*F*^2^), *S*	0.036, 0.084, 1.03
No. of reflections	4282
No. of parameters	199
H-atom treatment	H-atom parameters constrained
Δρ_max_, Δρ_min_ (e Å^−3^)	0.25, −0.38

Computer programs: *APEX3* and *SAINT* (Bruker, 2017 ▸), *SHELXT* (Sheldrick, 2015*a* ▸), *SHELXL* (Sheldrick, 2015*b* ▸), *ORTEP-3 for Windows* (Farrugia, 2012 ▸), *PLATON* (Spek, 2020 ▸) and *publCIF* (Westrip, 2010 ▸).

## supplementary crystallographic information

### 3-[(E)-2-(2-Bromo-4,5-dimethoxyphenyl)ethenyl]-5,5-dimethylcyclohex-2-en-1-one Crystal data

**Table:**

C_18_H_21_BrO_3_	*F*(000) = 752
*M**_r_* = 365.26	*D*_x_ = 1.405 Mg m^−^^3^
Monoclinic, *P*2_1_/*c*	Mo *K*α radiation, λ = 0.71073 Å
*a* = 11.9084 (8) Å	Cell parameters from 9892 reflections
*b* = 8.1667 (5) Å	θ = 2.6–24.9°
*c* = 18.1737 (13) Å	µ = 2.39 mm^−^^1^
β = 102.231 (2)°	*T* = 300 K
*V* = 1727.3 (2) Å^3^	Block, yellow
*Z* = 4	0.19 × 0.17 × 0.09 mm

### 3-[(E)-2-(2-Bromo-4,5-dimethoxyphenyl)ethenyl]-5,5-dimethylcyclohex-2-en-1-one Data collection

**Table:**

Bruker APEXII CCD diffractometer	2767 reflections with *I* > 2σ(*I*)
Radiation source: i-mu-s microfocus source	*R*_int_ = 0.045
φ and ω scans	θ_max_ = 28.3°, θ_min_ = 2.6°
Absorption correction: multi-scan (SADABS; Krause *et al.*, 2015)	*h* = −15→15
*T*_min_ = 0.636, *T*_max_ = 0.746	*k* = −10→10
33400 measured reflections	*l* = −24→24
4282 independent reflections	

### 3-[(E)-2-(2-Bromo-4,5-dimethoxyphenyl)ethenyl]-5,5-dimethylcyclohex-2-en-1-one Refinement

**Table:**

Refinement on *F*^2^	0 restraints
Least-squares matrix: full	Hydrogen site location: inferred from neighbouring sites
*R*[*F*^2^ > 2σ(*F*^2^)] = 0.036	H-atom parameters constrained
*wR*(*F*^2^) = 0.084	*w* = 1/[σ^2^(*F*_o_^2^) + (0.032*P*)^2^ + 0.5285*P*] where *P* = (*F*_o_^2^ + 2*F*_c_^2^)/3
*S* = 1.03	(Δ/σ)_max_ < 0.001
4282 reflections	Δρ_max_ = 0.25 e Å^−^^3^
199 parameters	Δρ_min_ = −0.38 e Å^−^^3^

### 3-[(E)-2-(2-Bromo-4,5-dimethoxyphenyl)ethenyl]-5,5-dimethylcyclohex-2-en-1-one Special details

**Table:**

Geometry. All esds (except the esd in the dihedral angle between two l.s. planes) are estimated using the full covariance matrix. The cell esds are taken into account individually in the estimation of esds in distances, angles and torsion angles; correlations between esds in cell parameters are only used when they are defined by crystal symmetry. An approximate (isotropic) treatment of cell esds is used for estimating esds involving l.s. planes.

### 3-[(E)-2-(2-Bromo-4,5-dimethoxyphenyl)ethenyl]-5,5-dimethylcyclohex-2-en-1-one Fractional atomic coordinates and isotropic or equivalent isotropic displacement parameters (Å^2^)

**Table:**

	*x*	*y*	*z*	*U*_iso_*/*U*_eq_	
Br1	0.21244 (2)	0.20268 (3)	0.03311 (2)	0.06997 (12)	
O1	0.66634 (15)	1.0834 (2)	0.14033 (11)	0.0836 (6)	
O2	−0.01107 (12)	0.74963 (19)	−0.16748 (9)	0.0603 (4)	
O3	−0.11576 (12)	0.47454 (18)	−0.16639 (9)	0.0600 (4)	
C1	0.52228 (18)	0.8950 (3)	0.09158 (12)	0.0569 (6)	
H1	0.477268	0.976060	0.063868	0.068*	
C2	0.63384 (19)	0.9412 (3)	0.13716 (12)	0.0586 (6)	
C3	0.70601 (18)	0.8051 (3)	0.17758 (13)	0.0554 (6)	
H3A	0.757898	0.849533	0.221475	0.067*	
H3B	0.752286	0.759373	0.144658	0.067*	
C4	0.63502 (16)	0.6684 (2)	0.20260 (11)	0.0441 (5)	
C5	0.54760 (17)	0.6074 (3)	0.13380 (12)	0.0511 (5)	
H5A	0.587780	0.544519	0.102108	0.061*	
H5B	0.493931	0.534370	0.150666	0.061*	
C6	0.48127 (17)	0.7415 (3)	0.08760 (11)	0.0471 (5)	
C7	0.37120 (18)	0.7062 (3)	0.03714 (11)	0.0536 (6)	
H7	0.335261	0.793272	0.008546	0.064*	
C8	0.31710 (17)	0.5639 (3)	0.02768 (11)	0.0496 (5)	
H8	0.352859	0.475488	0.055327	0.060*	
C9	0.20528 (16)	0.5337 (3)	−0.02282 (10)	0.0444 (5)	
C10	0.14791 (17)	0.3853 (3)	−0.02615 (11)	0.0452 (5)	
C11	0.04082 (17)	0.3600 (3)	−0.07330 (11)	0.0478 (5)	
H11	0.004492	0.259107	−0.073778	0.057*	
C12	−0.01094 (16)	0.4839 (3)	−0.11895 (11)	0.0452 (5)	
C13	0.04507 (16)	0.6359 (3)	−0.11840 (11)	0.0449 (5)	
C14	0.15029 (17)	0.6574 (3)	−0.07094 (11)	0.0478 (5)	
H14	0.186674	0.758185	−0.070673	0.057*	
C15	0.5738 (2)	0.7347 (3)	0.26188 (12)	0.0638 (6)	
H15A	0.523896	0.822936	0.240823	0.096*	
H15B	0.529205	0.648981	0.277875	0.096*	
H15C	0.629523	0.774031	0.304298	0.096*	
C16	0.7137 (2)	0.5289 (3)	0.23628 (15)	0.0737 (7)	
H16A	0.752552	0.486776	0.199091	0.111*	
H16B	0.769349	0.568486	0.278733	0.111*	
H16C	0.669031	0.443436	0.252311	0.111*	
C17	0.0350 (2)	0.9103 (3)	−0.16221 (16)	0.0733 (7)	
H17A	−0.011771	0.978807	−0.199475	0.110*	
H17B	0.036049	0.953757	−0.112985	0.110*	
H17C	0.111881	0.907246	−0.170618	0.110*	
C18	−0.1695 (2)	0.3181 (3)	−0.17712 (15)	0.0699 (7)	
H18A	−0.242412	0.327809	−0.211566	0.105*	
H18B	−0.121412	0.243649	−0.197319	0.105*	
H18C	−0.180950	0.277343	−0.129703	0.105*	

### 3-[(E)-2-(2-Bromo-4,5-dimethoxyphenyl)ethenyl]-5,5-dimethylcyclohex-2-en-1-one Atomic displacement parameters (Å^2^)

**Table:**

	*U*^11^	*U*^22^	*U*^33^	*U*^12^	*U*^13^	*U*^23^
Br1	0.07534 (19)	0.05863 (17)	0.06375 (17)	−0.00047 (12)	−0.01275 (12)	0.01078 (12)
O1	0.0711 (11)	0.0741 (12)	0.0917 (13)	−0.0346 (10)	−0.0141 (10)	0.0226 (10)
O2	0.0468 (9)	0.0514 (9)	0.0701 (10)	−0.0037 (7)	−0.0158 (8)	0.0076 (8)
O3	0.0446 (8)	0.0494 (9)	0.0727 (10)	−0.0071 (7)	−0.0174 (7)	−0.0094 (7)
C1	0.0484 (12)	0.0665 (15)	0.0475 (12)	−0.0123 (11)	−0.0084 (10)	0.0180 (11)
C2	0.0511 (13)	0.0709 (16)	0.0493 (13)	−0.0201 (12)	0.0006 (10)	0.0117 (11)
C3	0.0369 (11)	0.0714 (15)	0.0527 (13)	−0.0080 (11)	−0.0025 (9)	−0.0028 (11)
C4	0.0350 (10)	0.0482 (12)	0.0439 (11)	0.0024 (9)	−0.0034 (9)	−0.0009 (9)
C5	0.0413 (11)	0.0528 (13)	0.0534 (12)	−0.0024 (10)	−0.0030 (9)	−0.0061 (10)
C6	0.0389 (11)	0.0640 (13)	0.0338 (10)	−0.0089 (10)	−0.0022 (8)	0.0018 (9)
C7	0.0449 (12)	0.0648 (15)	0.0432 (11)	−0.0074 (11)	−0.0087 (9)	0.0096 (10)
C8	0.0426 (11)	0.0579 (13)	0.0431 (11)	−0.0016 (10)	−0.0025 (9)	−0.0001 (10)
C9	0.0379 (11)	0.0529 (12)	0.0381 (10)	−0.0046 (9)	−0.0020 (8)	−0.0038 (9)
C10	0.0443 (11)	0.0480 (12)	0.0388 (10)	0.0015 (10)	−0.0012 (9)	−0.0024 (9)
C11	0.0452 (12)	0.0455 (11)	0.0488 (12)	−0.0087 (10)	0.0011 (10)	−0.0084 (10)
C12	0.0375 (11)	0.0474 (12)	0.0454 (11)	−0.0022 (9)	−0.0033 (9)	−0.0099 (9)
C13	0.0373 (11)	0.0473 (11)	0.0458 (11)	−0.0017 (9)	−0.0011 (9)	−0.0018 (9)
C14	0.0396 (11)	0.0489 (12)	0.0496 (12)	−0.0090 (9)	−0.0024 (9)	−0.0016 (9)
C15	0.0647 (15)	0.0781 (16)	0.0462 (13)	0.0081 (13)	0.0062 (11)	0.0081 (12)
C16	0.0533 (14)	0.0646 (16)	0.0885 (18)	0.0120 (12)	−0.0180 (13)	0.0019 (14)
C17	0.0567 (15)	0.0519 (14)	0.099 (2)	−0.0056 (12)	−0.0103 (13)	0.0167 (14)
C18	0.0552 (14)	0.0580 (15)	0.0837 (18)	−0.0178 (12)	−0.0141 (13)	−0.0112 (13)

### 3-[(E)-2-(2-Bromo-4,5-dimethoxyphenyl)ethenyl]-5,5-dimethylcyclohex-2-en-1-one Geometric parameters (Å, º)

**Table:**

Br1—C10	1.904 (2)	C8—C9	1.469 (3)
O1—C2	1.222 (3)	C8—H8	0.9300
O2—C13	1.361 (2)	C9—C10	1.386 (3)
O2—C17	1.418 (3)	C9—C14	1.404 (3)
O3—C12	1.361 (2)	C10—C11	1.393 (3)
O3—C18	1.424 (3)	C11—C12	1.369 (3)
C1—C6	1.342 (3)	C11—H11	0.9300
C1—C2	1.458 (3)	C12—C13	1.408 (3)
C1—H1	0.9300	C13—C14	1.374 (3)
C2—C3	1.499 (3)	C14—H14	0.9300
C3—C4	1.526 (3)	C15—H15A	0.9600
C3—H3A	0.9700	C15—H15B	0.9600
C3—H3B	0.9700	C15—H15C	0.9600
C4—C16	1.519 (3)	C16—H16A	0.9600
C4—C15	1.523 (3)	C16—H16B	0.9600
C4—C5	1.531 (3)	C16—H16C	0.9600
C5—C6	1.499 (3)	C17—H17A	0.9600
C5—H5A	0.9700	C17—H17B	0.9600
C5—H5B	0.9700	C17—H17C	0.9600
C6—C7	1.460 (3)	C18—H18A	0.9600
C7—C8	1.322 (3)	C18—H18B	0.9600
C7—H7	0.9300	C18—H18C	0.9600
			
C13—O2—C17	117.28 (16)	C9—C10—Br1	121.60 (14)
C12—O3—C18	117.56 (17)	C11—C10—Br1	116.06 (16)
C6—C1—C2	123.3 (2)	C12—C11—C10	119.95 (19)
C6—C1—H1	118.3	C12—C11—H11	120.0
C2—C1—H1	118.3	C10—C11—H11	120.0
O1—C2—C1	120.9 (2)	O3—C12—C11	125.25 (18)
O1—C2—C3	122.6 (2)	O3—C12—C13	115.06 (18)
C1—C2—C3	116.4 (2)	C11—C12—C13	119.69 (17)
C2—C3—C4	113.08 (17)	O2—C13—C14	125.48 (19)
C2—C3—H3A	109.0	O2—C13—C12	115.52 (17)
C4—C3—H3A	109.0	C14—C13—C12	118.99 (19)
C2—C3—H3B	109.0	C13—C14—C9	122.85 (19)
C4—C3—H3B	109.0	C13—C14—H14	118.6
H3A—C3—H3B	107.8	C9—C14—H14	118.6
C16—C4—C15	109.19 (19)	C4—C15—H15A	109.5
C16—C4—C3	109.57 (18)	C4—C15—H15B	109.5
C15—C4—C3	109.23 (18)	H15A—C15—H15B	109.5
C16—C4—C5	109.85 (18)	C4—C15—H15C	109.5
C15—C4—C5	110.41 (17)	H15A—C15—H15C	109.5
C3—C4—C5	108.58 (17)	H15B—C15—H15C	109.5
C6—C5—C4	113.95 (18)	C4—C16—H16A	109.5
C6—C5—H5A	108.8	C4—C16—H16B	109.5
C4—C5—H5A	108.8	H16A—C16—H16B	109.5
C6—C5—H5B	108.8	C4—C16—H16C	109.5
C4—C5—H5B	108.8	H16A—C16—H16C	109.5
H5A—C5—H5B	107.7	H16B—C16—H16C	109.5
C1—C6—C7	119.1 (2)	O2—C17—H17A	109.5
C1—C6—C5	120.65 (18)	O2—C17—H17B	109.5
C7—C6—C5	120.26 (19)	H17A—C17—H17B	109.5
C8—C7—C6	127.1 (2)	O2—C17—H17C	109.5
C8—C7—H7	116.5	H17A—C17—H17C	109.5
C6—C7—H7	116.5	H17B—C17—H17C	109.5
C7—C8—C9	125.5 (2)	O3—C18—H18A	109.5
C7—C8—H8	117.2	O3—C18—H18B	109.5
C9—C8—H8	117.2	H18A—C18—H18B	109.5
C10—C9—C14	116.18 (17)	O3—C18—H18C	109.5
C10—C9—C8	123.18 (19)	H18A—C18—H18C	109.5
C14—C9—C8	120.64 (19)	H18B—C18—H18C	109.5
C9—C10—C11	122.34 (19)		
			
C6—C1—C2—O1	−178.3 (2)	C8—C9—C10—C11	178.66 (19)
C6—C1—C2—C3	3.2 (3)	C14—C9—C10—Br1	178.11 (15)
O1—C2—C3—C4	148.4 (2)	C8—C9—C10—Br1	−2.1 (3)
C1—C2—C3—C4	−33.1 (3)	C9—C10—C11—C12	0.5 (3)
C2—C3—C4—C16	174.69 (19)	Br1—C10—C11—C12	−178.78 (16)
C2—C3—C4—C15	−65.7 (2)	C18—O3—C12—C11	−8.8 (3)
C2—C3—C4—C5	54.7 (2)	C18—O3—C12—C13	171.6 (2)
C16—C4—C5—C6	−168.58 (19)	C10—C11—C12—O3	−178.98 (19)
C15—C4—C5—C6	71.0 (2)	C10—C11—C12—C13	0.6 (3)
C3—C4—C5—C6	−48.8 (2)	C17—O2—C13—C14	−9.0 (3)
C2—C1—C6—C7	−176.7 (2)	C17—O2—C13—C12	172.3 (2)
C2—C1—C6—C5	2.9 (3)	O3—C12—C13—O2	−2.6 (3)
C4—C5—C6—C1	21.4 (3)	C11—C12—C13—O2	177.80 (19)
C4—C5—C6—C7	−159.02 (19)	O3—C12—C13—C14	178.63 (19)
C1—C6—C7—C8	−178.2 (2)	C11—C12—C13—C14	−1.0 (3)
C5—C6—C7—C8	2.2 (4)	O2—C13—C14—C9	−178.3 (2)
C6—C7—C8—C9	178.9 (2)	C12—C13—C14—C9	0.3 (3)
C7—C8—C9—C10	−174.6 (2)	C10—C9—C14—C13	0.8 (3)
C7—C8—C9—C14	5.2 (3)	C8—C9—C14—C13	−179.09 (19)
C14—C9—C10—C11	−1.2 (3)		

### 3-[(E)-2-(2-Bromo-4,5-dimethoxyphenyl)ethenyl]-5,5-dimethylcyclohex-2-en-1-one Hydrogen-bond geometry (Å, º)

**Table:**

*D*—H···*A*	*D*—H	H···*A*	*D*···*A*	*D*—H···*A*
C8—H8···Br1	0.93	2.76	3.212 (2)	111
C17—H17*C*···O1^i^	0.96	2.58	3.495 (3)	159

Symmetry code: (i) −*x*+1, −*y*+2, −*z*.
